# Possible limits of calibrating reading charts with the Landolt ring: a microscopic study

**DOI:** 10.1186/s40662-022-00302-5

**Published:** 2022-08-15

**Authors:** Wolfgang Radner, Michael Radner, Barbara Daxer, Armin Ettl

**Affiliations:** 1 Karl Landsteiner University of Health Sciences, Dr. Karl-Dorrek-Straße 30, 3500 Krems, Austria; 2 Department of Ophthalmology, University Hospital St. Pölten, Dunant-Platz 1, 3100 St. Pölten, Austria; 3 Austrian Academy of Ophthalmology, Mollgasse 11, 1180 Vienna, Austria

**Keywords:** Near vision charts, Landolt rings, Near visual acuity, Print quality, Reading acuity, Sizes of optotypes

## Abstract

**Purpose:**

To evaluate microscopically whether the print quality and accuracy of sizing of Landolt ring near vision charts are adequate for the calibration of reading charts.

**Methods:**

Near vision charts with Landolt rings from Oculus GmbH (C-Test; Wetzlar, Germany), Precision Vision (Woodstock, IL) and the RADNER Charts were examined, as well as custom-made Landolt rings optimized for print quality. Microscopic investigations and measurements were performed by using a Huvitz HSZ 600 stereomicroscope (Nikon NIS Elements software) to evaluate the height of the Landolt rings, the thickness of the lines, and the width of the openings. The deviations from the mathematically correct values, which were calculated as given in the EN/ISO 8596 and by the International Council of Ophthalmology (ICO), were analyzed (calculated for a test distance of 40 cm).

**Results:**

All the near vision charts showed notable deficiencies in print quality and aberrations from the nominal values in the height, thickness of the lines, and width of the openings. The openings were too narrow, whereas the height and thickness of the lines were larger than the nominal values. Even the openings of Landolt rings optimized for print quality were not always within an acceptable 5% tolerance and need further improvement.

**Conclusion:**

This study reports inaccuracies in the heights, thicknesses of the lines, and widths of the openings of Landolt rings in all the near vision charts investigated. The extent of these inaccuracies excludes such near vision charts as reference tests for the calibration of reading charts. The x-height in relation to the visual angle still seems to be the most reliable method for standardizing the print sizes for reading charts.

## Background

Since there is an increasing need for well-standardized reading charts in research concerning refractive surgery, the “Near Vision and Accommodation Committee of the American-European Congress of Ophthalmology (AECOS)” has analyzed methods for investigating near vision and intermediate vision and has recommended ETDRS-format near vision charts and the RADNER Reading Charts [1]. In addition, as there is no international norm for near vision charts, the International Organization of Standards (ISO) has recently approved a proposal to establish an ISO standard for reading and near vision charts and has installed a working group. Together with the already existing standard of the International Council of Ophthalmology (ICO), such initiatives raise the question of how to achieve homologated reading charts and, in particular, how to calibrate reading charts [2–5].

The quality and calibration of vision tests using optotypes are important for clinical and research purposes. This is also true for near vision charts and reading charts as tests used in human subjects should be calibrated and manufactured in the best possible quality. For distance acuity, optotypes have to be calibrated with Landolt rings by means of a comparative psychophysical study in at least 10 participants that is given in the ISO/TR 19498:2015 [6]. Accordingly, optotypes can be deemed equivalent to the Landolt ring when the mean visual acuity of a group of at least 10 participants is within ± 0.05 log-units of the mean obtained with the Landolt ring, and when the standard deviation (SD) does not exceed 1.5 times the SD of the Landolt ring. However, it is unclear whether this or a similar method can also be applied to calibrating reading charts.

Modern reading charts are designed in accordance with the already existing standard of the ICO and use the x-height (height of a lower case x) of a type font [2–5]. The x-height must subtend a visual angle of 5 min of an arc as specified for a particular test distance (usually 40 cm) and geometrically increase (or decrease) by a factor of 10 to the power of 0.1. The Landolt ring was first introduced by Landolt in 1888 [7], and in 1907 it became the standard optotype at the Congress of the ICO in Naples [8]. Today, this standard is defined in the norm EN/ISO 8596 [9]. Landolt rings are constructed according to the principles postulated by Snellen for optotypes in 1864 [10]: (a) the height and width of an optotype have to subtend 5 min of an arc related to a specific test distance; (b) the thickness of the lines of which an optotype is constructed must be one-fifth of the optotype’s height, a rule that is (c) also valid for the blank opening of the Landolt ring, which also has to be one-fifth of the optotype's height. For manufacturing distance visual acuity charts with Landolt rings, the standard EN/ISO 8596 [9] and the standards postulated by the ICO [4] allow aberrations (tolerances) from the mathematically calculated nominal values of the geometrically progressing sizes. For EN/ISO 8596, the permissible tolerance is ± 5% until − 0.2 logarithm of the minimal angle of resolution (logMAR) and ± 10% for − 0.3 logMAR. In Chapter V of the ICO standard, a deviation of not more than ± 3% from the geometric progression is recommended, and a maximal acceptable tolerance of ± 5% is postulated for clinical purposes [4].

It is therefore evident, that lower case letters of a type font and Landolt rings differ in construction. Furthermore, the spacing between lower case letters is much smaller within words than between Landolt rings on a near vision chart. Thus, although both the x-height and the Landolt ring are of the same height as those are standardized to subtend 5 min of an arc for a specified test distance there are psychophysically relevant differences in construction and layout between reading charts and near vision charts using Landolt rings. Furthermore, only a small area of the fovea can be investigated with Landolt rings, which represents angular visual acuity (detail vision) [11], while for reading, a much larger area of the retina is involved. Reading charts investigate a functional aspect of vision that is of importance for our patients to accomplish necessities of everyday life (functional vision) [11]. Accordingly, it has been shown that visual acuity obtained with single optotype can significantly differ from reading acuity in several diseases [12, 13]. Thus, near vision charts using Landolt rings and reading charts investigate different psychophysical tasks that cannot unconditionally be compared.

Given that (a) Landolt rings in near vision charts and lower case letters of reading charts differ in construction, (b) according to EN/ISO 8596, the spacing between the Landolt rings is supposed to avoid crowding, which is a psychophysical aspect of reading charts [4, 9, 13], (c) distance vision and near vision are different visual tasks [11–14], and (d) it is unclear whether near vision charts with Landolt rings are available that qualitatively allow a valid psychophysical comparison, the suitability of using Landolt rings to calibrate reading charts remains questionable. Nevertheless, the key question is: are near vision charts available that provide Landolt rings of sufficient size and print quality to allow a valid and reliable psychophysical calibration? Therefore, this study was initiated to determine by means of a microscopic measuring system whether the print quality of commercially available near vision charts based on Landolt rings is sufficient for a valid calibration of reading charts.

## Methods

Commercially available near vision charts with Landolt rings were obtained from Oculus GmbH (C-Test; Wetzlar, Germany) and from Precision Vision (Woodstock, IL, USA). For each test, two versions were investigated. The C-Test consists of two charts: one in which the distances between adjacent Landolt rings (six Landolt rings per line) is 30 min of arc and another one in which the distance between adjacent Landolt rings is 2.6 min of arc (12 Landolt rings per line). The Landolt ring chart from Precision Vision presented five rings per line in ETDRS format. In addition, the Landolt rings represented on a near vision chart in the RADNER Reading Charts booklet were investigated (five Landolt rings per line), as well as a logarithmically progressing set of offset-printed, custom-made Landolt rings that had been graphically optimized in terms of height, thickness of the lines, and size of the openings by means of microscopical analyses. The C-Test, the Landolt rings of the RADNER Reading Charts, and the custom-made Landolt rings were printed with offset printing, and the Landolt rings of the near vision chart from Precision Vision were printed with screen printing.

**Fig. 1:**
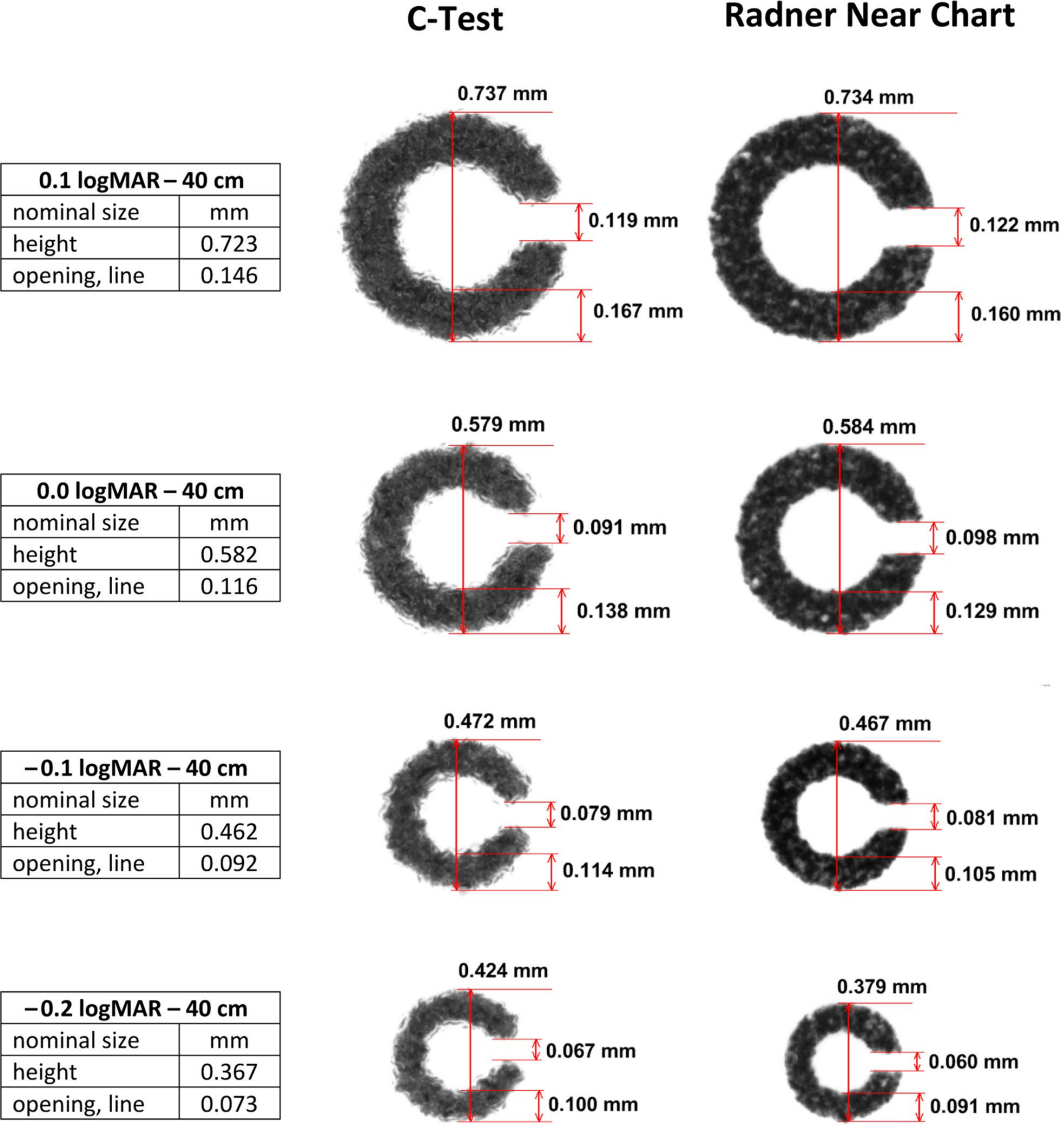
C-Test Landolt ring test (left column) and Landolt rings of the near vision chart from the RADNER Charts (right column); original magnification, ×40. Photographs of Landolt rings taken of print sizes ranging from 0.1 to − 0.2 logMAR. Nominal sizes are given between the columns. The C-Test shows deficiencies in print quality and aberrations in the optotype heights, thicknesses of the lines, and widths of the openings. The shape of the Landolt rings and endings that configure the opening of the Landolt rings are distorted. The lines are blurred and frayed. The openings are too narrow; the heights and thicknesses of the lines are > 5% compared with the nominal values. The Landolt rings of the near vision charts from the RADNER charts are more accurate. The edges of the lines are sharper, and the endings that form the openings are in parallel. The heights are within the 5% tolerance, but the widths of the openings and lines lie outside that tolerance

**Fig. 2:**
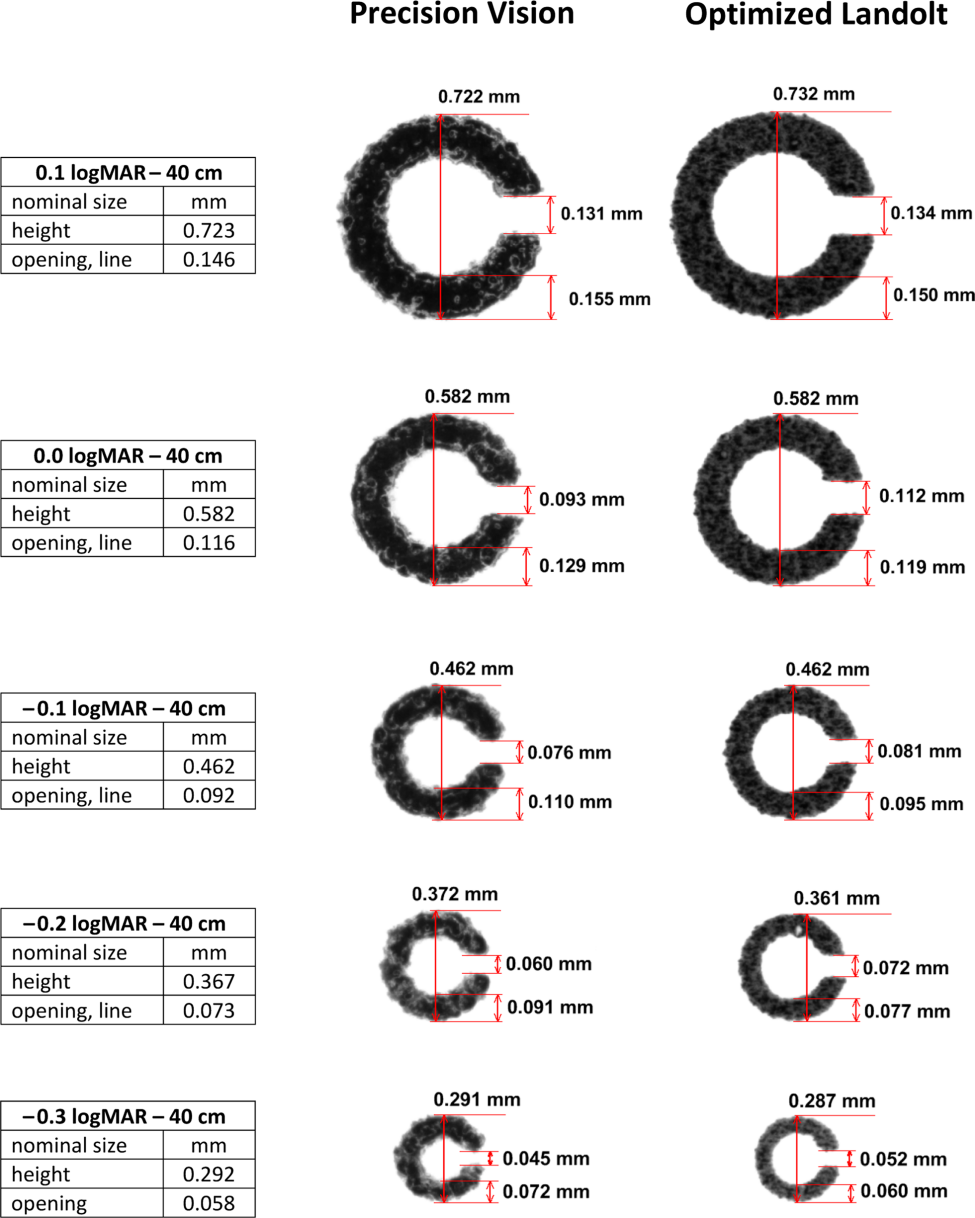
Landolt ring near vision chart from Precision Vision (left column) and Landolt rings graphically optimized for print quality (right column; RADNER optimized); original magnification, ×40. Photographs of Landolt rings taken of print sizes ranging from 0.1 to − 0.3 logMAR. Nominal sizes are given between the columns. The Landolt ring near vision charts of Precision Vision show deficiencies in print quality and aberrations in the optotype heights, thicknesses of the lines, and widths of the openings. The endings that configure the opening of the Landolt rings are parallel from 0.0 to 0.1 logMAR, but the lines are blurred. The heights of the Landolt rings are within the 5% tolerance at all sizes. The openings are narrower, and the widths of the lines are wider than the nominal values (outside 5%). The Landolt rings optimized for printed quality show the best print quality. The edges of the lines are sharp, and the endings at the openings are clearly parallel. The Landolt rings closely match the nominal values in height and line width. Only two openings are outside the 5% tolerance, i.e., − 0.1 and 0.1 logMAR

Microscopic analyses were performed using Nikon NIS Elements software with a 2.3 megapixel microscope color camera (Optocam III) mounted on a Huvitz HSZ 600 stereomicroscope (magnification 40×). The accuracy of microscopic measurements depends on the calibration, the number of pixels of the camera, and the aberrations of the optic. The measuring system was calibrated using a Zeiss stage micrometer. With this setup, the accuracy of the measurements was better than 0.5 µm. Measurements were performed in the still image mode of the NIS software using the cursor lines of the software. The distance in mm was directly shown on the screen. Figures 1 and 2 represent original micrographs that have not been graphically reworked. The following sizes of Landolt rings were measured for each near vision chart: (a) Landolt rings of the RADNER Reading Charts, from − 0.2 to 0.1 logMAR; (b) Oculus C-Test, from − 0.15 to 0.1 logMAR; (c) Precision Vision, from − 0.3 to 0.1 logMAR; (d) custom-made optimized Landolt rings, from − 0.3 to 0.1 logMAR.

The following quality-related characteristics were investigated: (a) print quality, (b) height of the Landolt rings (diameter), (c) thickness of the lines, and (d) width of the openings of the Landolt rings. The sizes of the Landolt rings were calculated for a test distance of 40 cm. Heights, line thicknesses, and widths of the openings were evaluated in terms of their degree of consistency with the nominal values, which were calculated based on the EN/ISO 8596 [9]. In addition, print quality was evaluated based on morphologic criteria by investigating the accuracy of print at the edges of the lines and the edges forming the openings of the Landolt rings (frayed or blurred edges, rounded edges of the openings).

## Results

Figures 1 and 2 show the print quality and measurements of the investigated near vision charts. At all print sizes, the Landolt rings of the offset printed C-Test samples exhibited deficiencies in print quality and considerable aberrations of the optotype heights, thicknesses of the lines, and widths of the openings (Fig. 1). The shapes of the Landolt rings were distorted, as were the endings that configure the opening of the Landolt rings. These endings appeared to be rounded and were not parallel to each other. In addition, the lines were blurred and frayed. The openings were too narrow, whereas the heights and thicknesses of the lines were larger than the nominal values and fell outside the allowed 5% tolerance (Table 1).

**Table 1:** Differences in percent from the calculated nominal values

Parameters	C-Test	Landolt-C RADNER charts	Landolt-C ETDRS format	Landolt-C custom made
0.1 logMAR				
Height	1.90	1.52	− 0.14	1.24
Opening	− 18.49	− 16.44	− 10.27	− 8.22
Line thickness	14.38	9.59	6.16	2.74
0.0 logMAR				
Height	− 0.52	0.34	0.00	0.00
Opening	− 21.55	− 15.52	− 19.83	− 3.45
Line thickness	18.97	11.21	11.21	2.59
− 0.1 logMAR				
Height	2.16	1.08	0.00	0.00
Opening	− 14.13	− 11.97	− 17.29	− 11.96
Line thickness	23.91	14.13	19.57	3.26
− 0.2 logMAR				
Height	15.23^a^	3.27	1.36	− 1.63
Opening	− 8.22^a^	− 17.81	− 17.81	− 1.47
Line thickness	36.99^a^	24.66	24.66	5.48
− 0.3 logMAR				
Height	–	–	− 0.44	− 1.71
Opening	–	–	− 22.41	− 10.44
Line thickness	–	–	24.14	3.45

^a^The original visual acuity grade of the C-Test is − 0.15 logMAR, which causes a bigger difference in the calculated nominal values for − 0.2 logMAR

The screen-printed Landolt ring near vision charts from Precision Vision showed similar inaccuracies, but to a lesser extent (Fig. 2). The Precision Vision Landolt rings appeared to be correctly round. At 0.0 and 0.1 logMAR, the endings at the openings were almost parallel (others were rounded). The heights of the Landolt rings were close to the nominal values and within the 5% tolerance at all sizes. However, the openings were narrower for − 0.3 to 0.1 logMAR, and the thicknesses of the lines were wider than the nominal values (outside the 5% tolerance) (Table 1).

The Landolt rings presented in the near vision charts of the RADNER Reading charts showed more accurate print quality (Fig. 1). The edges of the lines were sharper than those of the other two near vision charts, and the endings that form the openings were parallel. The heights were within the 5% tolerance. The aberrations of the openings and thicknesses of the lines were similar to those of the Landolt ring charts from Precision Vision but better than those of the C-Test (Table 1).

The custom-made, optimized, offset-printed Landolt rings showed the best print quality. The edges of the lines were sharp, and the endings at the openings were parallel. All the Landolt rings closely matched the height and line thickness of the nominal values (Fig. 2), and thus were well within the 5% tolerance. The openings for 0.0 and − 0.2 logMAR were within the 5% tolerance, those for − 0.3, − 0.1 and 0.1 logMAR were 10.44%, 11.96% and 8.22% narrower than the nominal values, respectively (Table 1).

## Discussion

For calibration of a psychophysical visual acuity test, it is indispensable that a reference test with Landolt rings be constructed accurately in accordance with the EN/ISO 8596 [9] and commercially available tests. However, we report here that commercially available near vision charts show inaccuracies in print quality and aberrations from the nominal values for the height, thickness of the lines, and size of the openings of the Landolt rings. Although the results obtained at smaller print sizes (smaller 0.1 logMAR) will not be comparable with angular visual acuity, these aberrations might not be of high relevance for routine clinical work but must be taken into account for research, as these might lead to artificial ceiling effects. The extent of these inaccuracies excludes such near vision charts as reference tests for a calibration.

For this study, we microscopically pre-selected the Landolt ring near vision charts with the best print quality. In other near vision charts, the smallest print size was only 0.0 logMAR for a test distance of 40 cm, and therefore not applicable for calibration. Screens of electronic devices could not be used because of an insufficient resolution at small print sizes [15].

Aberrations of offset print are due to an outflow of the color when it is pressed on the substrate (e.g., paper) between the rubber blanket cylinder and the pressure cylinder [16], or in case of screen print when the color goes beyond the edges of the stencil on the mesh [17]. Both aspects cannot be fully avoided and cause oversizing of lines and of the optotypes, as well as frayed or blurred edges, and rounded edges of the opening. For the RADNER Reading Charts, the print quality has been optimized with the printing company for text print. However, offset print is a technology that has been developed for text print, and thus is not exceptionally accurate for graphically constructed figures such as optotypes. We therefore made an attempt to optimize a set of Landolt rings for near vision charts by means of microscopic measurements and graphical modification. The custom made Landolt rings were constructed by a graphic designer. Lines of 8 Landolt rings were adjusted to the mathematically calculated nominal sizes from − 0.3 to 1.0 logMAR. These sets of Landolt rings were then printed by the printing company and the height of the Landolt rings, the thickness of the lines, and the width of the openings were then measured for every print size. The sizes were modified according to differences to the mathematically calculated values (e.g., when a parameter was too big, it was made smaller; when the opening was too small, it was increased). Then, the Landolt rings were printed again. This procedure has been performed in order to approach, step by step, the calculated nominal sizes on the print. Here, we used the set from the fourth round of modification (further modifications will follow). This level of improved accuracy gave values that were very close to the mathematically calculated nominal values for the diameter of the Landolt rings and the thickness of the lines. However, there were still openings of the Landolt rings that were too narrow and outside the 5% tolerance (− 0.2, − 0.1 and 0.1 logMAR). Although we believe that we can get even closer to the nominal sizes, these results confirm that manufacturing Landolt rings representing an appropriate reference for the calibration of near vision charts is technically much more complex than it is for distance vision, because the impact of the typical inaccuracies of print, such as oversizing, is much bigger in relation to the optotypes´ sizes (optotypes for distance acuity tested at 4 m are tenfold larger than those for near vision charts for a test distance of 40 cm).

Another concern is that reading charts and reference tests must cover a sufficient range of geometrically progressing print sizes. To avoid ceiling effects, the smallest print size for near vision charts with single optotypes should be − 0.3 logMAR, and for reading charts, − 0.2 logRAD (for a test distance of 40 cm; logRAD = reading equivalent of logMAR) [18]. However, the sizes of the optotypes of many near vision charts with Landolt rings only extend down to − 0.1 or 0.0 logMAR. Even the C-Test does not go down to − 0.2 logMAR. The smallest print size of this test is − 0.15 logMAR for a test distance of 40 cm. Although it might be clinically reasonable to use half of a geometric step at such a small print size, this approach and the limited sizing are not appropriate for the calibration of other tests.

In addition, we could not find a near vision chart that was entirely in accordance with the layout as required for the EN/ISO 8596 [9]. To avoid crowding effects, the EN/ISO 8596 requires that the horizontal and vertical distances between optotypes increase with smaller print size (e.g., from 0.0 to 0.4 logMAR these distances are two times the diameter of the largest Landolt rings displayed; for smaller sizes than 0.0 logMAR, they are three times the diameter) [9]. However, crowding is a phenomenon that is typical when words and sentences are read from reading charts [3, 4]. This situation raises two questions: (a) How should the Landolt rings be arranged on a near vision chart used for the calibration of reading charts? and (b) What is a calibration with the Landolt rings intended to compare?

Reading and recognizing details of optotypes do not represent the same visual tasks [11]. Colenbrander recommends that “reading tests that show how well the patients function, should not be ignored in routine medical practice. Because the goal of all medical interventions ultimately is to improve the functioning of the person” [11]. Furthermore, tests of reading vision tell us how the patients perform, and they represent the functional vision needed to accomplish the visual necessities of everyday life, whereas single optotype vision evaluation such as that carried out with Landolt rings only investigates a retinal area that is smaller than 1 degree from the foveal center at a visual acuity of 1.0 logMAR—what Colenbrander calls detail vision [11]. In addition, for clinical purposes, reading acuity has its own place in evaluating near visual performance and also its own notation. This situation is reasonable, given that single-optotype distance acuity has been shown to be a poor predictor of reading performance [14] and that in several eye diseases, it is typical that distance acuity and reading acuity differ significantly (e.g., age-related maculopathy and amblyopia) [12, 13]. Therefore, it seems evident that, independent of the technical limits of calibrating reading charts with Landolt rings, their usefulness for reading charts can also be questioned psychophysically.

It should also be mentioned that once calibration has been done based on the specifications outlined by the ISO/TR 19498:2015 [6], the x-heights of a reading chart will have to be adjusted according to the differences determined by this psychophysical study. However, such studies compare the means and standard deviations obtained from two geometrically progressing tests expressed in log units. Converting differences in geometric means into the linear system of x-heights in millimeters is complex and complicates manufacturing. We further report that the investigated Landolt ring near vision charts cannot even fulfill the criterion of a 5% tolerance because of the technical limits of print quality [4, 6].

Nevertheless, as long as a calibration with Landolt rings has not been realized, the ICO recommends the use of the x-height of a font and calibration of this height according to the visual angle of 5 min of an arc, as specified for the related test distance of the geometric progression [4].

The present study shows that near vision charts with Landolt rings do not achieve a level of quality sufficient to meet the premises for calibration. Since the x-height related to the visual angle represents an already well-recognized standard that has been demonstrated in many studies to produce reproducible and reliable results for reading acuity and other reading parameters [2, 3, 5], it seems to be obvious to retain this definition as the standard for reading charts in Latin script.

## Conclusion

The extent of the deficiencies in print quality and the inaccuracies in the heights, thicknesses of the lines, and widths of the openings of Landolt rings exclude Landolt ring near vision charts as a reference for the calibration of reading charts. The x-height of a font in relation to the visual angle, representing the current standard, is still the more reliable method for standardizing the print sizes of reading charts.

## Data Availability

Not applicable.

## Abbreviations

AECOS: American-European Congress of Ophthalmology
ETDRS: Early treatment diabetic retinopathy study
ICO: International Council of Ophthalmology
ISO: International Organization of Standardization
logMAR: Logarithm of the minimal angle of resolution
logRAD: Logarithm of the reading acuity determination. The logRAD is the reading equivalent of logMAR and defined as the logarithm base 10 of the visual angle (minutes of arc) that subtends one-fifth of the x-height at the standardized distance of 40 cm
